# Pool Testing for COVID-19: Suitable Splitting Procedure and Pool Size for India

**DOI:** 10.1017/dmp.2020.335

**Published:** 2020-09-10

**Authors:** Balram Rai, Anandi Shukla, Geetika Choudhary, Abhishek Singh

**Affiliations:** Department of Mathematical Demography and Statistics, International Institute for Population Sciences, Mumbai; Department of Statistics, Ramanujan School of Mathematical Sciences, Pondicherry University, Pondicherry

**Keywords:** COVID-19, India, pool size, pool testing, splitting procedure

## Abstract

**Objective:**

Coronavirus disease (COVID-19) has emerged as a global pandemic for public health due to the large scale outbreak, therefore there is an urgent need to detect the infected cases quickly and isolate them in order to suppress the further spread of the disease. This study tries to identify a suitable pool testing method and algorithm for COVID-19.

**Methods:**

This study tries to derive a general equation for the number of tests required for a pooled sample to detect every infected individual in the specific pool. The gain in pool testing over the normal procedure is quantified by the percentage of tests required compared to individual testing.

**Results:**

The percentage of tests required by the pool testing strategy varies according to the different splitting procedures, the size of the pooled sample, and the probability of an individual being infected in the population. If the probability of infection is 0.05, then for a pool size of 32, only 14 tests are sufficient to detect every infected individual.

**Conclusion:**

The number of tests required to detect infected individuals by using the pooling method is much lower than individual testing. This may help us with increasing our testing capacity for COVID-19 by testing a large number of individuals in less time with limited resources.

## INTRODUCTION

On December 19, 2019, the cases of novel coronavirus – severe acute respiratory syndrome coronavirus 2 (SARS-CoV-2)/coronavirus disease (COVID-19) – presenting with infected pneumonia started in Wuhan, Central China. It had a large-scale outbreak, which resulted in a pandemic. Hence, it became a public health concern all over the world.^1^ On February 12, 2020, the disease was renamed *COVID-19* by the World Health Organization (WHO); for consistency, COVID-19 will be used in this paper to refer to both the virus, SARS-CoV-2, and the disease. It was found to be caused by a zoonotic coronavirus (now officially named *SARS-CoV-2*) having similarities with SARS and Middle East respiratory syndrome (MERS) coronaviruses.^2^ The outbreak was announced as a Public Health Emergency of International Concern by the end of January 2020.^3^

According to a WHO situation report, globally, there are more than 19 million laboratory-confirmed cases and 0.7 million deaths (as of August 2020). About 215 countries and regions are affected by this pandemic.^4,5^ The WHO Director-General in a media briefing stated that if nations can detect, test, treat, isolate, trace, and mobilize their people in the response, the countries with fewer numbers of cases can prevent these cases from becoming clusters and growing those clusters into community transmission.^6^ According to the journal *Nature*, in much of the world, people exhibiting mild or no symptoms are unable to get tested, meaning that the actual number of cases could be much higher.^7^ There is a need for mass testing to check a higher number of individuals as much as possible. The higher number of tests will help us in the early detection of the infected cases of COVID-19 in the population and isolate them to reduce the magnitude of the outbreak due to COVID-19.

This disease also strikes many middle-income and lower-income countries. Due to the lack of proper medical facilities and available resources, they are not able to do early detection tests and hence not able to sustain the low transmission rate. In that scenario, pool testing can be a measure to curb further spread of disease. In the view of limited test kits available, pool testing may be an efficient way to increase the coverage of testing COVID-19 in the population.

The pool testing method, also recognized as a group testing method, is a process in which the samples (specimen, eg, blood or urine) are mixed into a pooled sample to test for a binary response. The negative result indicates that the group contains no diseased sample, and a positive result suggests the presence of at least 1 infected individual. This method is useful not only for the classification of the diseased,^8^ but also for the estimation of the prevalence of the infected in the population.^9^

Germany, in recent times, has become the torchbearer for this method as it expanded its testing capacity without compromising the high quality of diagnosis.^10^ Germany has conducted around 8.5 million tests (as of August 6, 2020). Regarding the total number of tests performed for COVID-19 for various countries (as of August 6, 2020), we found that the United States (62 million), Russia (29 million), Brazil (13 million), Spain (7 million), and the United Kingdom (17 million) have conducted quite a high number of tests to identify infected individuals.^5^

India is a highly populated country with a densely populated population. COVID-19 has a high human-to-human transmissibility. Hence, India might be at a higher risk of sudden outbreaks as compared with any other country with less population density. As of now, there is no vaccine for this disease available; hence, the only feasible precautionary measure that comes in the picture is the early detection of non-asymptotic cases and isolating them to check further transmission of the disease. As of now, India has conducted around 22 million tests as of August 5, 2020, of which 2 million individuals have been confirmed positive.^11^ The number is just a small fraction of the Indian population. India requires high testing rates for COVID-19 to confirm suspected cases and isolate them to reduce the risk of a high transmission rate. The pooled method will be cost-effective, as well as time-saving. If mass testing is conducted using a pooled method, it will also be useful in flattening the curve. By this method, a higher number of people can be tested by a fewer number of kits, in less time. In this study, we have tried to find the optimum pool size, which can reduce the number of tests to a greater extent.

## METHODOLOGY

**FIGURE 1:**
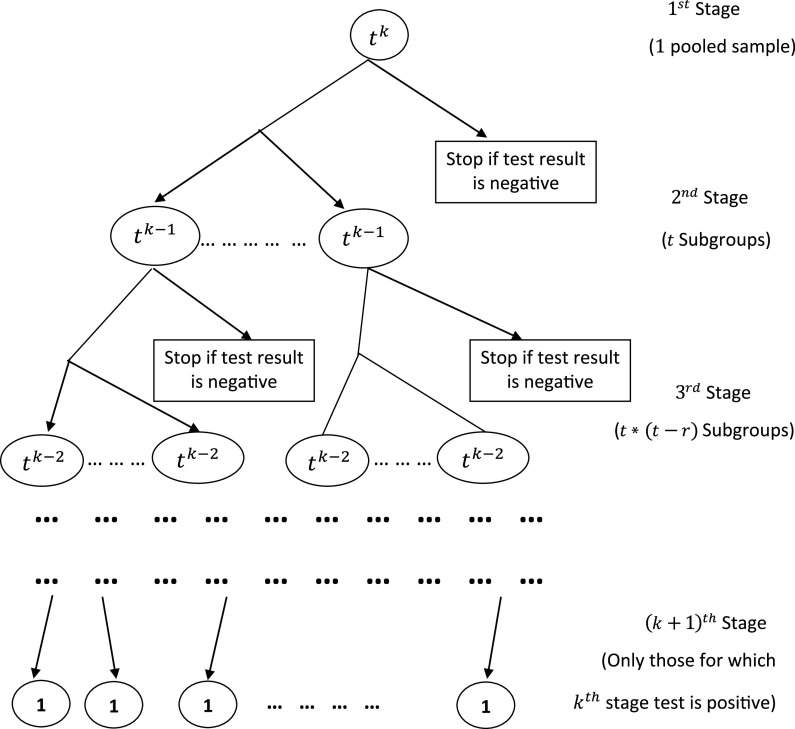
Flow Diagram for the Pool Size of the Form 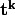.

Let us assume that the total number of individuals’ samples taken for testing at a time is of the form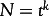. Here 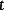 and 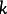 are positive integers (Figure 1).

**FIGURE 2:**
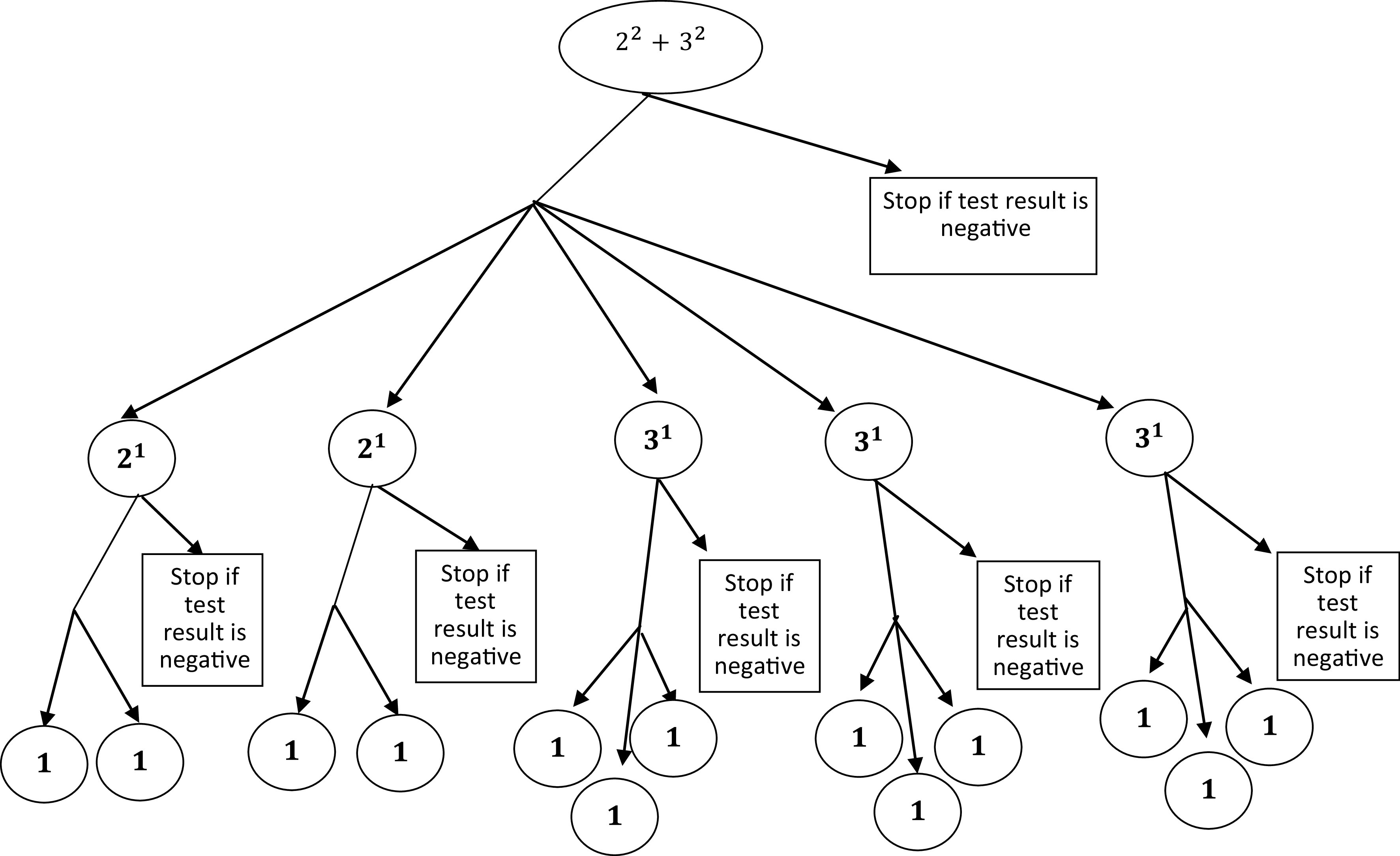
Flow Diagram for Mixed Strategy When *N* = 13.

Let 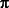 be the probability that an individual is infected with the disease, and we assume that every individual is independent of the other. This probability can be assigned by the experimenter with some previous knowledge or experience.

For our pool testing scenario, we mix all the samples and perform the test. It will have 2 outcomes, either positive or negative. Let us call this *stage* 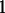.

The probability that the test result is negative at this stage will be
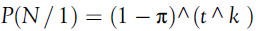



So, the probability that the test results in a positive, indicating that at least 1 individual in the sample is infected, will be:
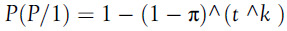



If the test result turns out to be negative, then we stop at that point and conclude that all the 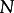 individuals are non-diseased. Whereas, if the test result is positive, then we split our sample into 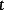 subgroups, each with 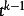 individual samples and then perform the test in 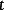 subgroups. We will call this *stage* 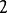.

The probability that the test result is negative at the 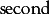 stage is shown as:
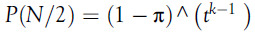



So, the probability that the test results in a positive at this stage will be:
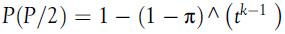



Likewise, we proceed further until we get all negative test results or we reach the 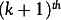 stage, where a subgroup contained only an individual’s sample. This procedure followed for the pooled testing is given below in an algorithm.

### Algorithm

- Take 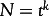 pooled sample at the first stage. Perform the test; it will have 2 outcomes: positive or negative.
- If the test in stage 1 turns out to be negative, then none of the individuals in the sample is infected; otherwise, divide the entire sample into 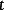 subgroups, and each will have 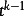 samples. Again, carry out the test for these 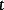 subgroups.
- Discard the subgroups whose results are negative and continue the procedure for the other subgroups. Suppose that 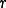 subgroups turn out to be negative, then there will be 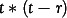 subgroups with each of size 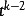 at stage 3.
- This procedure will continue until all subgroups at a particular stage test negative or up to the 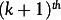 stage, where a subgroup contains only 1 individual.

The expected number of tests to detect all the diseased cases, denoted by 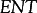, can be given by the following:
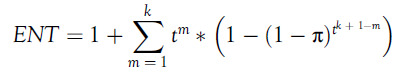



In particular, suppose 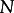 is of the form 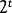. Let’s say there are 16 individuals, so 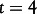 in this case. Then there will be maximum 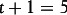 stages, at which we need to carry out the test.

The 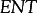 in this case will be 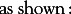


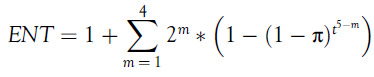



### Gain in Pooled Testing Over the Normal Procedure

The percentage of the expected number of tests 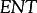 over the 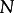 will give the percentage required to test if we adopt the pooled procedure over testing every individual.

### Mixed Strategy

Since if the 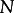 is of the form 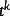, we have limited positive integers to select the pool size. For example, we cannot choose numbers such as 11, 14, 19, 23. The mixed kind of strategy will provide us with the wide range of positive integers for the selection of the pool size 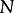.

We can also use a mixed strategy for testing. Let a sample size take the form 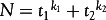. At stage 2, the pooled sample (stage 1 sample) can be divided into 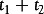 subgroups where 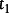 and 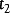 subgroups contain 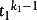 and 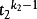 individuals, respectively. Afterward, we can proceed in usual steps described in the algorithm. The 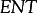 will be given by:
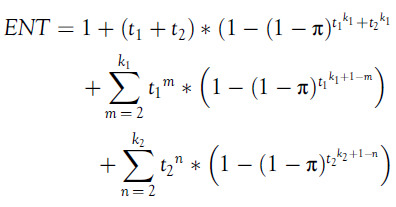



For example, if we take 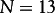, which is of the form, 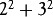, then the following flow diagram (Figure 2) will demonstrate the scenario.

### Data

The data for the number of tests done and for positive cases for COVID-19 in India have been derived from the update of the Indian Council of Medical Research on April 11, 2020. A total of 179 374 samples have been tested as of April 11, 2020, of which 7703 individuals have been confirmed positive among suspected cases and contacts of known positive cases in India.^12^

## RESULTS

### Results for Varying π

Table 1 provides the percentage of tests required by pool testing individuals as compared with testing every individual for different values of t, k, and π. The values of π have been taken as 0.001, 0.01, 0.02, and 0.05, representing the probability of an individual being infected in the population. The value of π depends on the population on which testing is required, which can vary among the population groups. The percentage of the required test indicates the percentage of tests that can be performed to detect every infected individual in a pool. If the π takes the value 0.05, then in a pool size of 32, only 42.6% of tests (ie, around 14 tests) are sufficient to detect every infected individual instead of carrying out 32 tests for all the individuals. Similarly, for a pool size of 64, only 44% tests (ie, around 28 tests) are sufficient to detect every infected individual in the sample. As the value of π increases (ie, the probability of an individual being infected in the population increases), the number of tests required for detecting the infected cases also increases for a fixed pool size. For a fixed t and π, the percentage of the required tests by using the pooling method also varies for different values of k, that is, distinct pool sizes.

**TABLE 1:** Percentage of the Required Tests by Pooling for Different Values of π, t, and k

	K	π Pool size	0.001	0.01	0.02	0.05
**t = 2**	1	2	50.2	52.0	54.0	59.8
	2	4	25.4	29.0	32.8	44.0
	3	8	13.1	18.4	24.1	39.9
	4	16	7.0	14.0	21.3	40.7
	5	32	4.1	12.6	21.1	42.6
	6	64	2.8	12.5	21.8	44.0
	7	128	2.2	12.9	22.5	44.8
	8	256	1.9	13.2	22.9	45.2
**t = 3**	1	3	33.6	36.3	39.2	47.6
	2	9	11.7	17.0	22.5	37.7
	3	27	4.6	12.2	19.8	38.6
	4	81	2.4	11.8	20.3	39.8
	5	243	1.9	12.1	20.7	40.2
**t = 4**	1	4	25.4	28.9	32.8	43.5
	2	16	7.0	13.9	20.9	38.8
	3	64	2.7	12.2	20.8	40.1
	4	256	1.9	12.5	21.1	40.5
**t = 5**	1	5	20.5	24.9	29.6	42.6
	2	25	5.0	13.3	21.5	41.1
	3	125	2.3	13.0	22.0	41.9

### Results for Indian Scenario

We have tried to analyze the results for India by applying the method of pool testing proposed in this study. The number of confirmed cases in India has been rising exponentially from the past few weeks. The rise in the number of COVID-19 cases has created an alarming situation for public health authorities in India. Though India has increased the number of tests for identifying the infected individual, being the world’s second-most populous country, there is a need for mass testing of the population for the early detection of cases and isolating them to stop the further spread of COVID-19.

The value of π for India comes out to be 0.04294 by taking the number of tests performed and the total confirmed positive cases till April 11, 2020. Table 2 provides the results for the number of tests required by the method of pool testing over testing every individual in the population. The number of tests required by using the pooling method varies for different pool sizes. For t = 2, the minimum percentage of testing required is for the pool size of 8 and 16, which indicates that only 3 tests are required by pool testing to detect every infected individual for a pool size of 8. Similarly, only 6 tests are sufficient for testing a pool size of 16 individuals. For t = 3, the minimum percentage of testing required is for the pool size of 9, indicating that only 3 tests are sufficient for testing a pool of 9 individuals. The minimum percentage of tests required for t = 4 and t = 5 is for the pool size of 16 and 25, respectively. Among all the pool sizes, the minimum percentage of tests required in the case of India is for the pool size of 9 (ie, 34.3%), which can be even more practically feasible compared with larger pool sizes.

**TABLE 2:** Results for Test Pooling in Case of the Present Scenario in India

			π = 0.04294	
	**k**	**Pool size**	**No. of tests required**	**Percentage of tests required**
**t = 2**	1	2	1	58.4
	2	4	2	41.5
	3	8	3	36.4
	4	16	6	36.4
	5	32	12	38.0
	6	64	25	39.4
	7	128	51	40.2
	8	256	104	40.5
**t = 3**	1	3	1	45.7
	2	9	3	34.3
	3	27	9	34.6
	4	81	29	35.8
	5	243	88	36.2
**t = 4**	1	4	2	41.1
	2	16	6	35.0
	3	64	23	36.2
	4	256	94	36.5
**t = 5**	1	5	2	39.7
	2	25	9	37.0
	3	125	47	37.8

**FIGURE 3:**
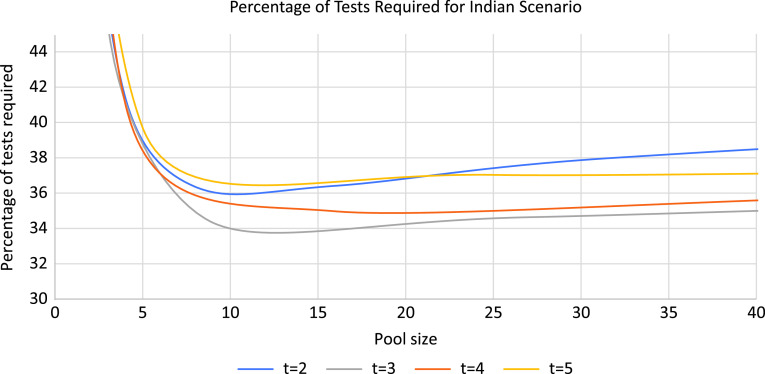
Percentage of Tests Required for Different Values of t in the Case of India.

Figure 3 illustrates the percentage of tests required for different values of t in the case of India. For t = 3, the percentage of the required testing by the pooling method is lower for almost all pool sizes as compared with other values of t.

## DISCUSSION

The results revealed that the percentage of tests required by the pool testing strategy varies according to different splitting procedures, size of the pooled sample, and the probability of an individual to be infected in the population. The optimum pooling strategy and pool size (N = t^k^) can be decided based on the information on the probability of an individual to be infected by COVID-19 in the population and the minimum number of tests required by the pooled sample. The general equation for the expected number of tests provides a method of simulations, which can be used to obtain the pool size (N = t^k^). For instance, we assume that the probability of an individual to be infected is constant in the population on which COVID-19 test has to be performed. The splitting procedure and the pool size can be chosen in such a way that it requires a minimum number of tests and is practically feasible. The pooling of samples largely depends on practical or laboratory feasibility also. The results showed that the number of tests required to detect the infected individuals by using the pooling method is much lower than the individual testing. This may help us in increasing our testing capacity for COVID-19 with testing a large number of individuals in less time with limited resources. The main advantage of pool testing is that if the pool tests negative, then we don’t need to test each individual separately. Even though the pool tests positive, the required number of tests required to detect the infected individuals is much lower as compared with individual testing. The pool testing strategy can also be useful in estimating the true prevalence of COVID-19 in the community by performing a large number of tests by pooling the individual samples.^13^ Recent studies have also suggested the asymptomatic transmission of COVID-19,^14,15^ in which case the pool testing method can be an important tool to identify such infected individuals. Sensitivity and specificity of the test also have an impact on pool testing.^16^ The sensitivity and specificity of the the test should be high in order to capture the true positive and true negative cases effectively.

In the case of India, the tertiary splitting procedure and a pool size of 9 were most suitable. A pool size of 9 will require only 3 tests for detecting every infected individual in the pooled sample. The value of π = 0.04294 was taken in view of the present scenario in India. If the government has to go for mass testing, the value for π is expected to decrease, which will reduce the number of testing even more as compared with the present scenario. There is significant regional variation in the probability of infection in Indian states, like Masharstra (0.19), Tamil Nadu (0.09), Delhi (0.12), Andhra Pradesh (0.08), and Uttar Pradesh (0.03).^12^ However, the optimum pooling strategy and pool size can be decided separately for these regions, based on the information of the infection rate. In mass testing, the government can also opt for a larger pool size to increase the coverage of testing to a greater extent. The pool testing strategy may help us with testing more individuals in less time in order to detect the infected individuals.

### Limitations

The pool testing method also has some limitations, which are essential to mention. One of the main limitations of the methodology described in this paper is that it assumes 100% sensitivity for the tests detecting COVID-19. However, evidence suggests that reverse transcriptase polymerase chain reaction (RT-PCR), which is the most commonly used test for COVID-19, has 100% sensitivity with small pool sizes.^17^ This method is more suitable and appropriate in low prevalence areas because, as the prevalence increases, the efficiency of the pool testing strategy declines. The other limitation of this study is the assumption of the constant probability of infection among individuals, which may not be true in all cases. Using the general derived equation, we can calculate the optimum pool size for different scenarios.
